# Supplementary material for: Indexing cerebrovascular health using near-infrared spectroscopy
Source: Sci Rep. 2021 Jul 20;11:14812. doi: 10.1038/s41598-021-94348-5 (PMC8292377; doi:10.1038/s41598-021-94348-5)
Supplement: Supplementary file 1 — Supplementary Information 1. [file 41598_2021_94348_MOESM1_ESM.pdf]

Supplementary Materials

Indexing Cerebrovascular Health Using Near-infrared Spectroscopy

Rashid Afkhami[1], Frederick R. Walker[2], Saadallah Ramadan[3], Rachel Wong[2], Sarah J. Johnson[1]

[1]University of Newcastle, School of Electrical Engineering & Computing
[2]University of Newcastle, School of Biomedical Sciences & Pharmacy
[3]University of Newcastle, School of Health Sciences

Estimation of CRF

Methods based on [Jurca et al., 2005]

Step 1: Acquiring physical activity score

**Physical Activity Description**	**Score**
Inactive or little activity other than usual daily activities	0.00
Regularly ($\geq$5 d/wk) participate in physical activities requiring low levels of exertion that result in slight increase in breathing and heart rate for at least **10 minutes** at a time	0.32
Participate in aerobic exercises such as brisk walking, jogging or running, cycling, swimming, or vigorous sports at a comfortable pace or other activities requiring similar levels of exertion for **20 to 60 minutes** per week	1.06
Participate in aerobic exercises such as brisk walking, jogging or running at a comfortable pace, or other activities requiring similar levels of exertion for **1 to 3 hours** per week	1.76
Participate in aerobic exercises such as brisk walking, jogging or running at a comfortable pace, or other activities requiring similar levels of exertion for **over 3 hours** per week	3.03

Step 2: Estimating CRF

$$\begin{aligned}\text{CRF} = &+ 2.77 \times (0 \text{ for women, } 1 \text{ for men}) \\ &- 0.10 \times (\text{Age in years}) \\ &- 0.17 \times \left(\text{Body mass index in kg/m}^2\right) \\ &- 0.03 \times (\text{Resting heart rate in beats per minute}) \\ &+ 1.00 \times (\text{Physical activity score}) \\ &+ 18.07\end{aligned}$$

[Jurca et al., 2005] Jurca, R. et al. (2005). Assessing cardiorespiratory fitness without performing exercise testing. *American Journal of Preventive Medicine*, 29(3):185–193.
